# Increased intestinal permeability and bile acid accumulation via inhibition of the FXR-SHP pathway contribute to coumarin-induced systemic inflammation

**DOI:** 10.1128/spectrum.01415-25

**Published:** 2025-09-19

**Authors:** Na Shou, Dandan Wu, Qi Wang, Ping Huang, Qiwen Lin, Senao Hou, Keyi Fu, Wenqian Xu, Jiyu Zhang, Zunji Shi

**Affiliations:** 1 State Key Laboratory of Herbage Improvement and Grassland Agro-ecosystems, Center for Grassland Microbiome, College of Pastoral Agriculture Science and Technology, Lanzhou University 12426, Lanzhou, China; University of Nebraska-Lincoln, Lincoln, Nebraska, USA

**Keywords:** coumarin, gut microbiota, LPS biosynthesis genes, bile acids, *Fxr-Shp *pathway

## Abstract

Coumarin causes severe poisoning in animals, affecting the development of agriculture and animal husbandry. Coumarin induces hepatotoxicity, but the inflammatory mechanism of coumarin is unclear. Here, we explored the effects of coumarin on systemic and hepatic inflammation. Our study found that coumarin reduced the abundance of *Akkermansia muciniphila*. The number of colonic goblet cells and the expression of *Claudin 1* and *ZO-1* were decreased in coumarin-treated groups, increasing intestinal permeability. Coumarin elevated the expression of lipopolysaccharide (LPS) biosynthesis genes and serum LPS. These changes increased oxidative stress indicators and total BA (TBA) in the liver. Coumarin inhibited *Fxr* and *Shp* expressions in the liver, thereby promoting cholesterol 7-alpha hydroxylase (*Cyp7a1*) transcription and upregulating TBA level. Collectively, coumarin induced systemic and hepatic inflammation by decreasing tight junction protein and colonic goblet cells, increasing LPS biosynthesis genes of gut microbiota and serum LPS, and elevating TBA level via the *Fxr-Shp* pathway.

**IMPORTANCE:**

Coumarin, a compound found in *Melilotus officinalis*, a high-quality forage plant crucial to animal husbandry, has raised safety concerns due to its potential for severe animal poisoning and liver toxicity. It is not clear about the mechanism of coumarin-induced systemic inflammation and liver inflammation. This research aims to elucidate the mechanisms of coumarin-induced systemic and hepatic inflammation, which is of significant importance for the development of agriculture and animal husbandry. In agriculture, understanding how coumarin affects the gut microbiota and intestinal barrier function could lead to the development of new varieties of *Melilotus officinalis* with lower coumarin concentrations, thus improving its safety as a forage crop.

## INTRODUCTION

*Melilotus officinalis (M. officinalis*) was considered an important forage and green manure crop, with broad applications in medicine and soil restoration (1). Coumarin, the secondary metabolite of *M. officinalis*, was recognized as a phytochemical with a variety of biological and pharmacological activities (2). Although coumarin had broad prospects for application in medical and agrochemical fields (3), they were also proven to cause severe poisoning or death in animals (4). Therefore, the concentrations of coumarin were usually considered in feed production (5). In animal models, coumarin has been shown to induce toxicity, including hepatocyte damage (6). This limited the clinical application of coumarin and the forage application of *M. officinalis*. Therefore, further study of the mechanism of coumarin-induced hepatotoxicity is very meaningful for the safe use of coumarin and the innovation and utilization of new varieties of *M. officinalis* with low concentrations of coumarin.

The liver, as a primary organ for drug metabolism, was critical in the process of coumarin metabolism. The metabolism of coumarin in the liver was mainly carried out by the cytochrome P450 enzyme system, which could produce electrophilic reactive intermediates during this process (7). These intermediates were capable of reacting with large molecules within cells, leading to cellular damage (8). Liver inflammation was an important stage in the development of toxicity, not only affecting the function of hepatocytes but also potentially triggering systemic inflammatory responses through the release of inflammatory mediators (9). The gut-liver axis further modulates this process, as disruptions in gut microbiota homeostasis can exacerbate liver inflammation. Lipopolysaccharides (LPS) are key components of the cell wall of gram-negative bacteria and play a significant role in mediating inflammation. Alterations in LPS biosynthetic genes can impact the stability and function of the gut microbiota (10), leading to elevated serum LPS levels and metabolic dysfunction (11). When the intestinal barrier function was compromised, LPS could more easily cross the intestinal barrier into the bloodstream (12). LPS could also induce liver immune cells to produce interleukin-1β (IL-1β), interleukin 6 (IL-6), tumor necrosis factor α (TNF-α), and other inflammatory factors and trigger oxidative stress, further promoting liver inflammation (13–15). In animal models, the injection of LPS could mimic systemic inflammation, including the exacerbation of liver inflammation, which may be associated with dysbiosis of the gut microbiota (16).

Changes in intestinal permeability were not only indicators of liver diseases but also potentially significant factors in driving disease progression (17). An imbalance in the gut microbiota led to the impairment of tight junctions in the intestinal barrier, thereby increasing intestinal permeability (18). When the intestinal permeability increased, bacteria or their metabolic products in the gut could more easily cross the intestinal barrier into the portal venous circulation, thereby affecting the liver and triggering or exacerbating liver inflammation (19). Goblet cells in the gut could secrete sticky proteins that formed the mucosal barrier (20). A decrease in goblet cells was often associated with the impairment of intestinal barrier function (21). A reduction in tight junction proteins such as *Occludin*, *Claudin 1*, and *ZO-1* also reflected the impairment of the integrity of the intestinal barrier (22). In the ileum, the gene expression level of the protein tyrosine phosphatase receptor H type (*Ptprh*) was a key biological indicator for assessing the health condition of intestinal permeability (23). When the intestinal barrier function was impaired, the composition of gut microbiota changed, characterized by a decrease in beneficial bacteria and an increase in harmful bacteria (24). An increase in harmful bacteria could lead to more bacterial metabolic products, such as LPS, entering the bloodstream, exacerbating systemic inflammation and liver inflammation (18).

Bile acids (BAs) were produced from cholesterol in the liver and transported to the intestine via the bile ducts. After microbial transformation in the gut, these bile acids cycled back to the liver and played a key role in the gut-liver axis. These bile acids were essential for maintaining intestinal homeostasis and alleviating inflammation (25). However, excessive bile acids disrupted the integrity of the intestinal barrier and activated immune cells in the liver, releasing pro-inflammatory factors, thereby exacerbating local and systemic inflammation (26). An increase in the level of BAs in serum was closely related to liver inflammation (27). The Farnesoid X receptor (FXR) played a key role in regulating the level of BAs in the enterohepatic circulation, inflammatory responses, and lipid and glucose homeostasis (28). Since *Fxr* activation was generally anti-inflammatory, the suppression of *Fxr* could amplify the inflammatory response (29). The *Fxr-Shp* signaling pathway, composed of the nuclear receptor *Fxr* and its direct target protein “small heterodimer partner” (*Shp*), could regulate the level of BAs and BA homeostasis (30). *Shp* was an inhibitor of cholesterol 7-alpha hydroxylase (*Cyp7a1*) expression, which was the rate-limiting enzyme in BA synthesis (31). In the ileum, the activation of *Fxr* induced the expression of fibroblast growth factor 19 (*Fgf19*) or fibroblast growth factor 15 (*Fgf15*) (32). Under cholestatic conditions, the elevated level of BAs induced hepatocytes to secrete cytokines, and hepatocytes could recruit neutrophils to initiate an inflammatory response (33).

In the past few years, the literature on coumarin had only touched upon hepatotoxicity, and most studies were not in-depth. It is not clear about the mechanism of coumarin-induced systemic inflammation and liver inflammation. Therefore, we investigated the effects of coumarin on the gut microbiota and its derivatives BAs and the *Fxr-Shp* signaling pathway through metagenomic sequencing, targeted metabolomics analysis, ELISA analysis, and quantitative real-time polymerase chain reaction (qPCR) analysis. Understanding these effects could provide valuable insights into the hepatotoxic mechanisms of coumarin, which will be of significance for the development of safer coumarin-based pharmaceuticals and agricultural products. In agriculture, understanding how coumarin affects the gut microbiota and intestinal barrier function could lead to the development of new varieties of *M. officinalis* with lower coumarin concentrations, thus improving its safety as a forage crop.

## MATERIALS AND METHODS

### Materials and reagents

Coumarin (≥98.5%) was obtained from Innochem Co., Ltd (Shanghai, China). Methanol (MeOH) (≥99.0%), HPLC-grade water (≥100%), and acetonitrile (MeCN) (≥99.99%) were purchased from Thermo Fisher Scientific Co., Ltd (Waltham, MA, USA). Isoflurane (≥98.0%) was obtained from Biogoethe Biotechnology Co., Ltd (Wuhan, China).

### Animals and treatment

The research ethics approval was included in the Supporting Information, and animal testing satisfied the ethical standards of the Lanzhou University College of Pastoral Agriculture Science and Technology ethics committee. In this study, male C57BL/6J mice (~23 g, about 6 weeks of age) were tested. The animals were maintained in a controlled environment with stable humidity (50% ± 5%), temperature (22 ± 1°C), and a light-dark cycle of 12 h each. After a week of adaptation, the mice were randomly divided into four groups, each group of eight. Coumarin was prepared in corn oil for administration. Although the control group received corn oil only, the experimental groups were administered varying concentrations of coumarin: the low dose group (5 mg/kg), the middle dose group (25 mg/kg), and the high dose group (125 mg/kg). The toxicological effects of coumarin have been widely studied in animals. In animal studies, a dose of 10 mg/kg body weight per day was considered safe for rats (34). However, higher doses, like 50 mg/kg body weight per day, can cause liver damage and other harmful effects. Similarly, it was reported that the minimum dose for hepatotoxicity was 25 mg/kg body weight (35). In this study, we chose 5 mg/kg body weight to represent the lowest level of coumarin exposure, 25 mg/kg body weight to study possible sub-toxic effects at a critical level, and 125 mg/kg body weight to explore the toxic effects at a higher level. Gavage was given every 2 days for 5 weeks. Mice in each group were allowed to eat and drink freely. The weight and chow consumption of the mice were measured weekly. After the mice were treated with isoflurane, serum, ileum, liver, and cecum contents were collected and stored at −80°C for the following experiments.

### Histochemical assessment of colonic goblet cells

To analyze the number of colonic goblet cells, proximal colonic tissue fragments were collected and fixed in 10%  formalin solution at room temperature for at least 24 h. The tissue was treated with periodic acid-Schiff (PAS) and then stained. Sections were imaged using confocal microscopy, and goblet cells of the colon were counted.

### Hepatic H&E staining

The liver tissues fixed with formalin were embedded in paraffin wax, and then, the sections were prepared. After staining with hematoxylin-eosin (H&E), photos were taken using the Nikon U-III multi-point sensing system (Nikon Corporation Co., Ltd., Tokyo, Japan).

### Quantitative real-time polymerase chain reaction analysis

RNA was isolated from liver and ileum tissues (100  mg) using an RNA isolation kit (Takara Biomedical Technology Co., Ltd, Beijing, China). cDNA was synthesized with 1 µg RNA using the Takara reverse transcription kit. The reaction system was performed with SYBR Green real-time PCR reagent by ABI StepOne™ (Applied Biosystems Co., Ltd, Foster City, CA, USA). The qPCR conditions were established as follows: 95°C for 20 s, then 95°C for 30 s, with β-actin as the reference, 60°C for 30 s, a total of 40 cycles. The qPCR data were analyzed using the ΔΔCT method. The primer sequences for *Fxr*, *Shp*, *Cyp7a1*, *Fgf15*, and *Ptprh*, fibroblast growth factor receptor (*Fgfr4*), tight junction proteins including *Claudin 1, Occludin,* and *ZO-1*, and *Akkermansia muciniphila (A. muciniphila*) are detailed in the Supporting Information, Table S1.

### Immunofluorescence analysis of ZO-1 protein

Frozen sections (8 µm) of the colon from all samples were fixed in 2% paraformaldehyde for 10 min and then washed three times in PBS. The cells were incubated with 200 µL blocking buffer (10% donkey serum in PBS plus 0.1% triton) for 1 h. The primary antibody, polyclonal rabbit anti-ZO-1 (Zymed Laboratories Co., Ltd., San Francisco, CA, USA), was incubated overnight at 4°C. After rinsing in PBS three times, the fluorescently labeled secondary antibodies were incubated at 37°C for 1 h, washed three times in PBS for 20 min, then fixed with aquapolymount and lid. The single-color channel fluorescence photos were reverse decolorized with red as a positive signal and black as a background. The ratio of the total number of red positive cells and tissue area represented the red positive cells density of ZO-1 protein.

### Metagenomic analysis of gut microbiota

DNA kit (Biogoethe Biotechnology Co, Ltd, Wuhan, China) was used to extract total DNA from cecal contents (200 mg) according to the instructions. DNA concentration and purity were detected by 1% agarose gel electrophoresis to ensure DNA integrity and extraction quality. A metagenomic DNA library was constructed according to Illumina manufacturer’s instructions and then sequenced on the Illumina NovaSeq platform. The original reads of metagenomic sequencing were treated with fastp v0.20.0, and high-quality reads were selected by quality control and removal of cohesion sequences. Host sequences were removed using BWA-MEM (v.0.7.17). For gene prediction, taxonomy, and functional annotation, open reading frames (ORFs) in contigs were identified using MetaGene (https://metagene.nig.ac.jp/metagene/download.html). Reads after quality control were mapped to the non-redundant gene catalog with 95% identity using SOAPaligner (https://mirror.sysu.edu.cn/kali/pool/main/s/soapaligner/, version 2.21), and gene abundance in each sample was evaluated.

### Quantification of BA metabolites

BAs from liver and serum were extracted, including the following categories: taurocholic acid (TCA), taurochenodeoxycholic acid (TCDCA), tauroursodeoxycholic acid (TUDCA), taurodeoxycholic acid (TDCA), tauro-α-muricholic acid (T-α-MCA), tauro-β-muricholic acid (T-β-MCA), deoxycholic acid (DCA), lithocholic acid (LCA), cholic acid (CA), chenodeoxycholic acid (CDCA), α-muricholic acid (α-MCA), and β-muricholic acid (β-MCA). TBA in the liver and serum was tested using the TBA test kit (JianCheng, Nanjing, China) according to the instructions. All samples were randomized, and quantitative determination of BAs was performed using UHPLC-QQQ-MS (Agilent Technologies Co., Ltd., Santa Clara, CA, USA). The conditions were set with mobile phases A (ultrapure H_2_O with 0.005% formic acid) and B (HPLC acetonitrile with 0.05% formic acid). Negative multiple reaction monitoring (MRM) mode was employed for mass detection, and quantification of BAs was performed using integrated peak areas and calibration curves. A total of 28 standards of BAs were procured from Steraloids Co., Ltd (Newport, RI, USA). Information on BA metabolites is shown in Supporting Information (Table S2).

### ELISA analysis

Limulus lysate (LAL) assay (Shanghai Huyu Biotechnology Co., Ltd., Shanghai, China) was used to detect the concentration of free-form LPS in serum (Munford, 2016). ELISA kits (Shanghai Huyu Biotechnology Co., Ltd., Shanghai, China) were used to detect the serum proinflammatory factors, including IL-6, IL-1β, and TNF-α.

### Oxidative stress index measurement

The contents of catalase (CAT), malondialdehyde (MDA), superoxide dismutase (SOD), and total antioxidant capacity (T-AOC) were determined by corresponding kits (Biogoethe Biotechnology Co., Ltd, Wuhan, China).

### Statistical analysis

R software and cloud tools (https://www.bioincloud.tech) were used for α-diversity and β-diversity analysis. GraphPad software (version 8.0, USA) was used for graphic description and statistical interpretation. The random forest analysis was performed to identify the most important features contributing to the classification of samples using the Weka Bioincloud platform (36). One-way ANOVA or two-tailed Student’s *t* test was used to explain the differences between pairwise comparisons, and *P* < 0.05 was considered statistically significant.

## RESULTS

### Coumarin increased intestinal permeability

**Fig 1:**
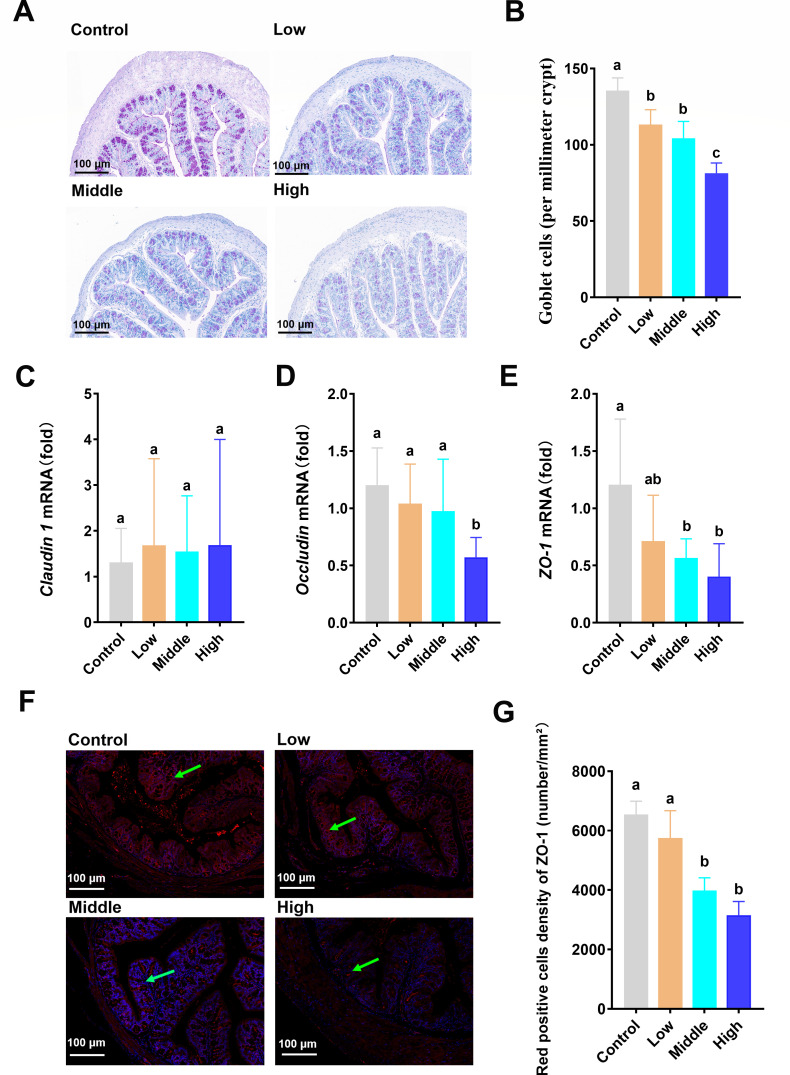
The alteration of intestinal permeability by coumarin. (**A**) Periodic acid-Schiff (PAS) staining for colonic tissue. Scale, 100 µm. (**B**) The number of colonic goblet cells. (**C–E**) The mRNA expression of *Claudin 1*, *occludin*, and *ZO-1* by qPCR. (**F**) Immunofluorescence analysis of tight junction protein *ZO-1*. The red fluorescence indicated by the green arrow represents ZO-1 areas in the intestine. Scale bars in panels, 100 µm. (**G**) Red positive cells density of ZO-1 in the intestine. Data are presented as the means ± standard deviation (SD) with a sample size of *n* = 8 per group. Statistical analysis was performed using one-way ANOVA, followed by a post-hoc test to determine significant differences among groups. Different letters above the bars indicate significant differences between groups at the *P* < 0.05 level.

The number of goblet cells in the colon was markedly decreased in the coumarin-treated group (Fig. 1A and B). Quantitative PCR was used for the detection of *Claudin 1*, *Occludin,* and *ZO-1* mRNA expression (Fig. 1C through E). There was no significant difference in the mRNA expression of *Claudin 1* between pairwise comparisons (Fig. 1C). *Occludin* mRNA expression was significantly decreased by a high dose of coumarin (Fig. 1D). Compared with the control and low groups, *ZO-1* mRNA expression was significantly reduced in the middle and high groups (Fig. 1E). Immunofluorescence analysis of tight junction protein ZO-1 exhibited that the fluorescence area of ZO-1 was reduced in the middle and high groups (Fig. 1F). The density of red positive cells of the ZO-1 protein in the middle and high doses was remarkably reduced compared with that in the control and low groups (Fig. 1G).

### Coumarin altered the intestinal microbial community

**Fig 2:**
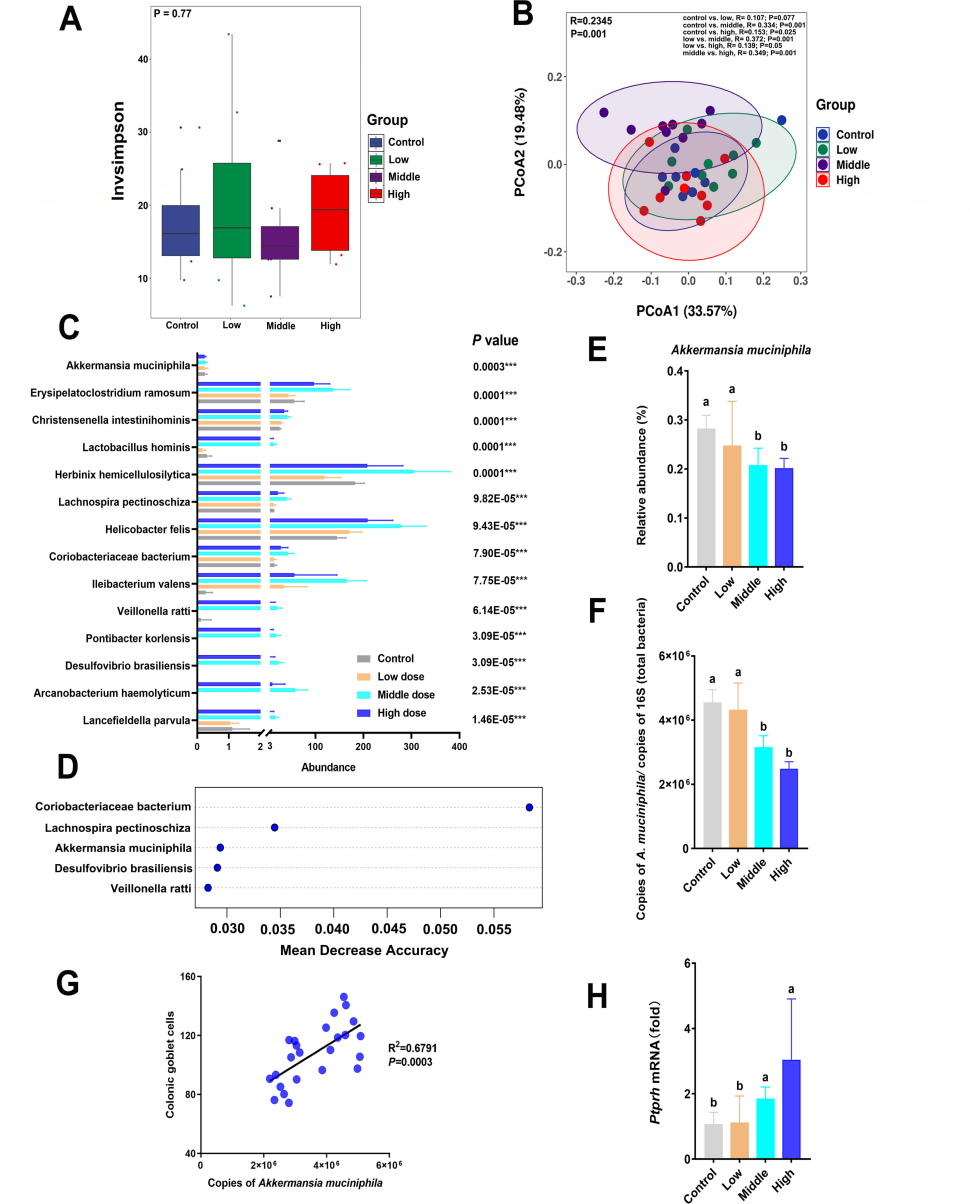
Analysis of gut microbial diversity and *A. muciniphila* abundance. (**A**) α-diversity differences based on the invsimpson index. (**B**) β-diversity differences based on the Bray-Curtis matrix by PCoA. (**C**) The significantly different bacterial species were identified through the Kruskal-Wallis test. *** *P* < 0.001. (**D**) The top five significant differential species identified by random forest. (**E**) The abundance of *A. muciniphila* based on metagenomic analysis. (**F**) The quantitative analysis of *A. muciniphila*. (**G**) Correlation scatter plot between colonic goblet cells and *A. muciniphila* species. (**H**) The expression level of *Ptprh* mRNA. Data are presented as the means ± SD with a sample size of *n* = 8 per group. Statistical analysis was performed using one-way ANOVA, followed by a post-hoc test to determine significant differences among groups. Different letters above the bars indicate significant differences between groups at the *P* < 0.05 level.

The intestinal bacterial community composition was analyzed based on metagenomic sequencing. The α-diversity analysis showed no significant difference among groups (Fig. 2A). The β-diversity analysis exhibited significant differences among different groups (*P* = 0.001) (Fig. 2B), which was verified by analysis of similarities (ANOSIM) analysis (control vs. low, *P* = 0.077; control vs. middle, *P* = 0.001; control vs. high, *P* = 0.025; low vs. middle, *P* = 0.001; low vs. high, *P* = 0.05; middle vs. high, *P* = 0.001). At the phylum level, Firmicutes, Verrucomicrobia, Bacteroidetes, and Proteobacteria were the most abundant bacterial phyla (Fig. S1A). At the genus level, *Akkermansia* was the most dominant genus (Fig. S1B). The Kruskal-Wallis test revealed that 14 species of bacteria were significantly different among the four groups (Fig. 2C, *P* < 0.001 and *q* < 0.05). To identify the key bacterial species associated with coumarin-induced gut dysbiosis, we performed random forest analysis based on differential bacterial species (Fig. 2D). *Coriobacteriaceae bacterium*, *Lachnospira pectinoschiza*, *A. muciniphila*, *Desulfovibrio brasiliensis*, and *Veillonella ratti* were identified as the top contributors to the model’s predictive accuracy, as indicated by their high mean decrease accuracy values (Fig. 2D). It is notable that the middle and high doses of coumarin significantly reduced the abundance of *A. muciniphila* compared with the control and low dose groups (Fig. 2E and F). We further found a strong and positive correlation (R^2^ = 0.6791, *P* < 0.0003) between the number of colonic goblet cells and the copies of *A. muciniphila* (Fig. 2G). The expression of *Ptprh* mRNA, a biomarker of intestinal barrier permeability, was significantly increased in the middle and high doses of coumarin compared with the control and low dose groups (Fig. 2H).

### Coumarin increased LPS biosynthetic genes in the gut and serum LPS content

**Fig 3:**
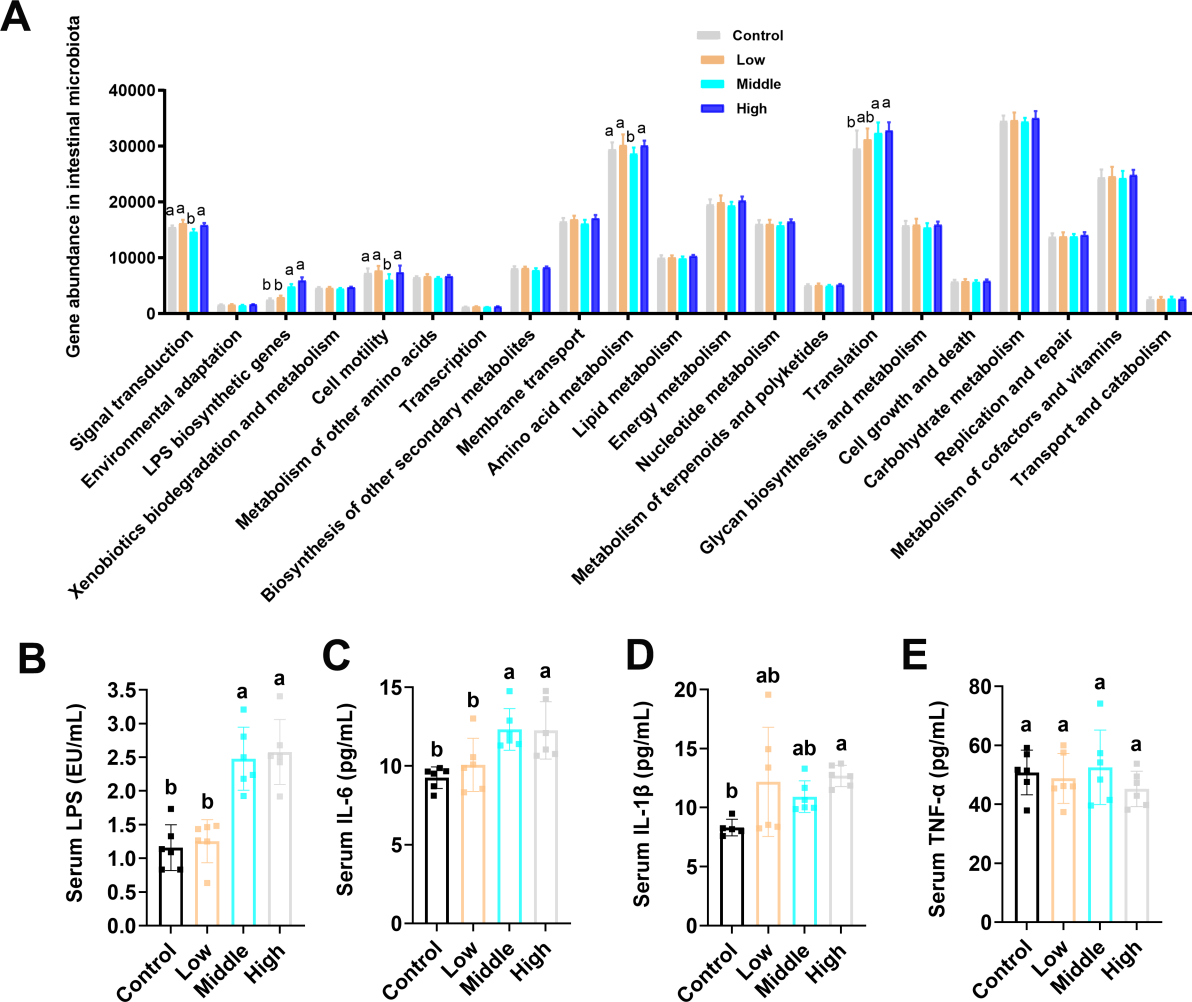
Analysis of LPS biosynthetic genes in the gut and serum LPS. (**A**) Metagenomic functional analysis of LPS biosynthetic genes. (**B**) Concentration of serum LPS. (**C**) The content of serum IL-6 was measured using ELISA. (**D**) The content of serum IL-1β was measured using ELISA. (**E**) The content of serum TNF-α by ELISA. Data are presented as the means ± SD with a sample size of *n* = 8 per group. Statistical analysis was performed using one-way ANOVA, followed by a post-hoc test to determine significant differences among groups. Different letters above the bars indicate significant differences between groups at the *P* < 0.05 level.

In contrast to the control and low dose groups, the middle and high doses of coumarin significantly increased the abundance of “LPS biosynthetic genes” (Fig. 3A). The middle dose of coumarin was observed to significantly decrease the gene abundance in the pathways of “Signal transduction,” “Cell motility,” and “Amino acid metabolism” (Fig. 3A). The gene abundance of “Translation” was significantly increased in the middle and high groups compared with the control group (Fig. 3A). Serum LPS content was markedly increased in the middle and high dose groups compared with the control and low groups (Fig. 3B). Serum IL-6 level in the middle and high dose groups was markedly increased (Fig. 3C), and serum IL-1β level in the high dose group was notably enhanced in comparison to the control group (Fig. 3D). Coumarin had no significant impact on serum TNF-α level (Fig. 3E).

### Coumarin induced hepatic inflammation by enhancing oxidative stress response and increasing TBA level

**Fig 4:**
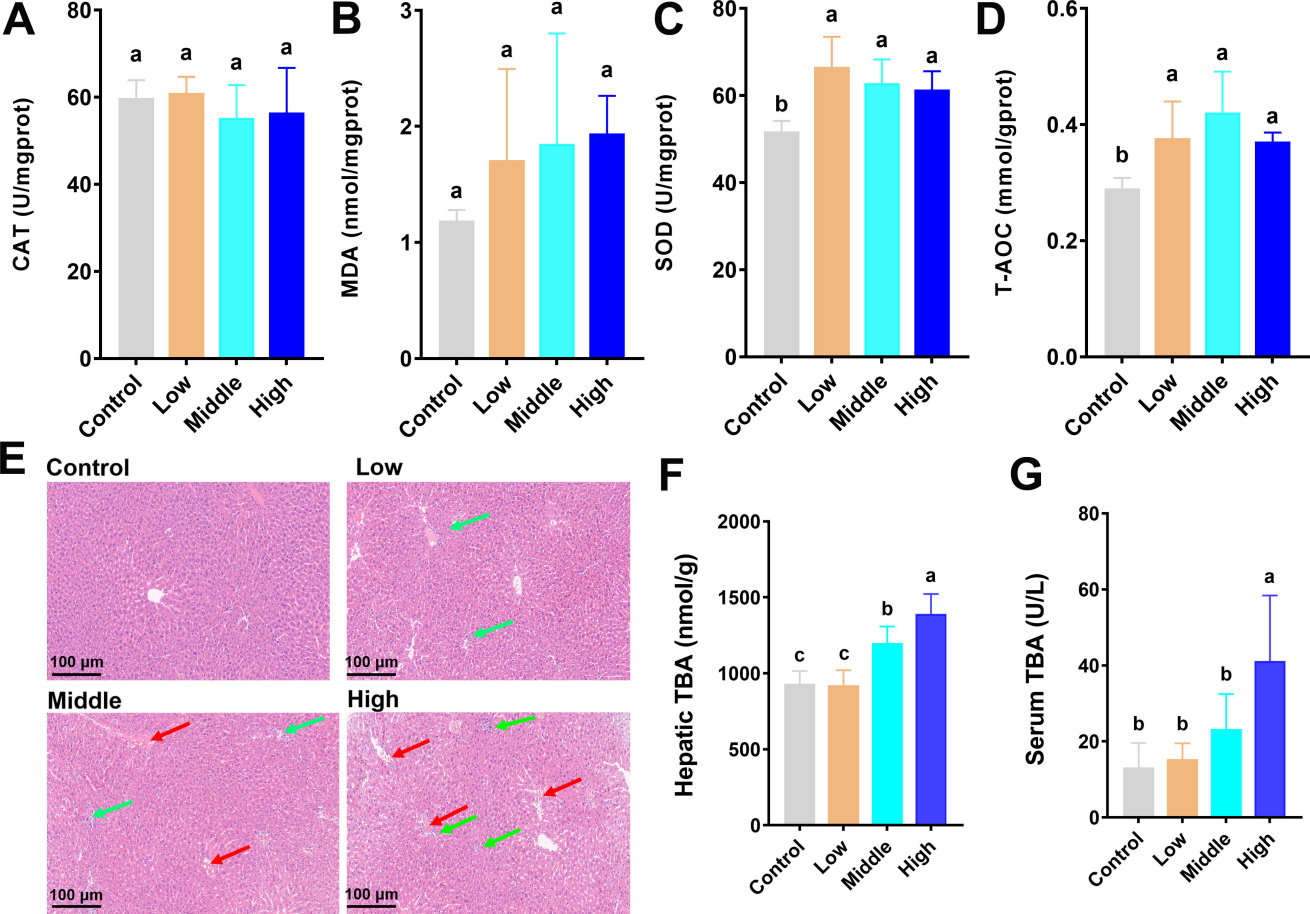
The oxidative stress response by coumarin in the liver. (**A**) The activity of hepatic CAT. (**B**) The activity of hepatic MDA. (**C**) The activity of hepatic SOD. (**D**) The activity of hepatic T-AOC. (**E**) Microscopic images (×400) of H&E-stained liver tissues. Blue arrows indicate the nuclear condensation areas of hepatocytes. Red arrows indicate the inflammatory cell infiltration areas of hepatocytes. (**F**) The content of hepatic TBA. (**G**) The content of serum TBA. Data are presented as the means ± SD with a sample size of *n* = 8 per group. Statistical analysis was performed using one-way ANOVA followed by a post-hoc test to determine significant differences among groups. Different letters above the bars indicate significant differences between groups at the *P* < 0.05 level.

We determined the oxidative stress in the liver of mice treated with different doses of coumarin. There were no significant changes in CAT and MDA after treatment with coumarin (Fig. 4A and B). In the middle and high dose groups, there was a notable elevation in SOD activity in comparison to the control group (Fig. 4C). The T-AOC activity was markedly elevated in the coumarin-treated group compared to the control group (Fig. 4D). The results of H&E staining demonstrated that the coumarin could induce the inflammatory cell infiltration and nuclear condensation of hepatocytes compared with the control group (Fig. 4E). Furthermore, our findings suggested that the hepatic TBA in the middle and high dose groups was markedly enhanced in contrast to the control and low dose groups (Fig. 4F). Similarly, serum TBA level was notably elevated in the high dose group than in the other groups (Fig. 4G).

### Coumarin disrupted the BA pool in the serum and liver

**Fig 5:**
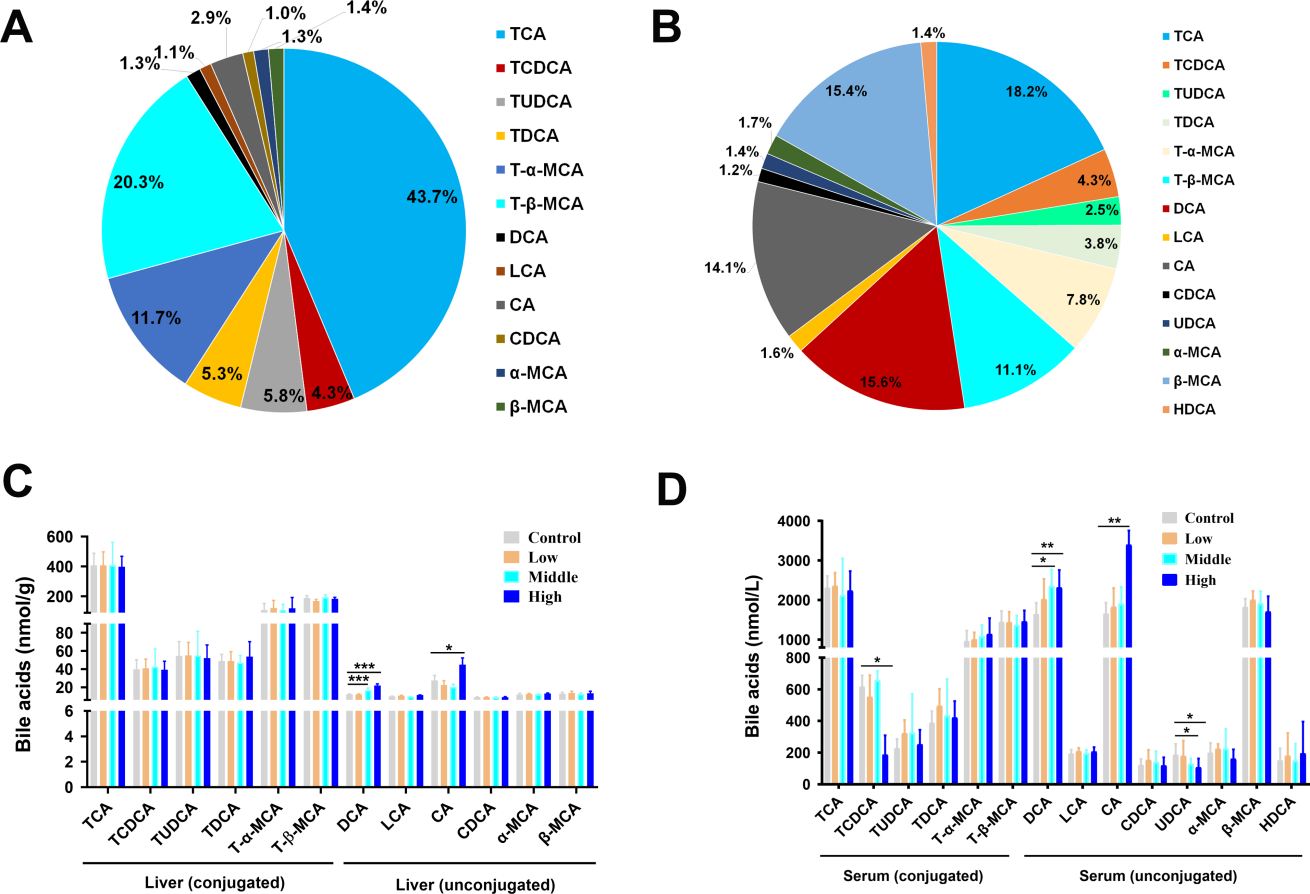
UHPLC-QQQ-MS quantification of unconjugated and conjugated BAs in serum and liver. (**A**) Percentage of hepatic BAs in the control group. (**B**) Percentage of serum BAs in the control group. (**C**) Concentrations of unconjugated and conjugated BAs in the liver. (**D**) Concentrations of unconjugated and conjugated BAs in the serum. Data are the means ± SD (*n* = 8) using one-way ANOVA. * *P* < 0.05, ** *P* < 0.01, *** *P* < 0.001.

In the hepatic BA pool of the control group, the conjugated BAs (TCA, 43.7%; T-β-MCA, 20.3%; T-α-MCA, 11.7%) were the most prevalent BAs, based on targeted quantification of BAs (Fig. 5A). The conjugated BAs (TCA, 18.2%; T-β-MCA, 11.1%) and the unconjugated BAs (CA, 14.1%; DCA, 15.6%; β-MCA, 15.4%) in the serum BA pool of the control group were dominant BAs (Fig. 5B). In the liver, the DCA was significantly increased in the middle and high doses of coumarin, and the CA was markedly upregulated in the high dose of coumarin (Fig. 5C). In the serum, the TCDCA was significantly reduced in the high dose group, the DCA in the middle and high dose groups was significantly higher than that in the control group, and the CA in the high dose group was remarkably higher than that in the control group (Fig. 5D). The UDCA in serum was significantly reduced in the middle and high dose groups than in the control group (Fig. 5D).

### Coumarin increased hepatic TBA by inhibiting the *Fxr***-***Shp* signaling pathway

**Fig 6:**
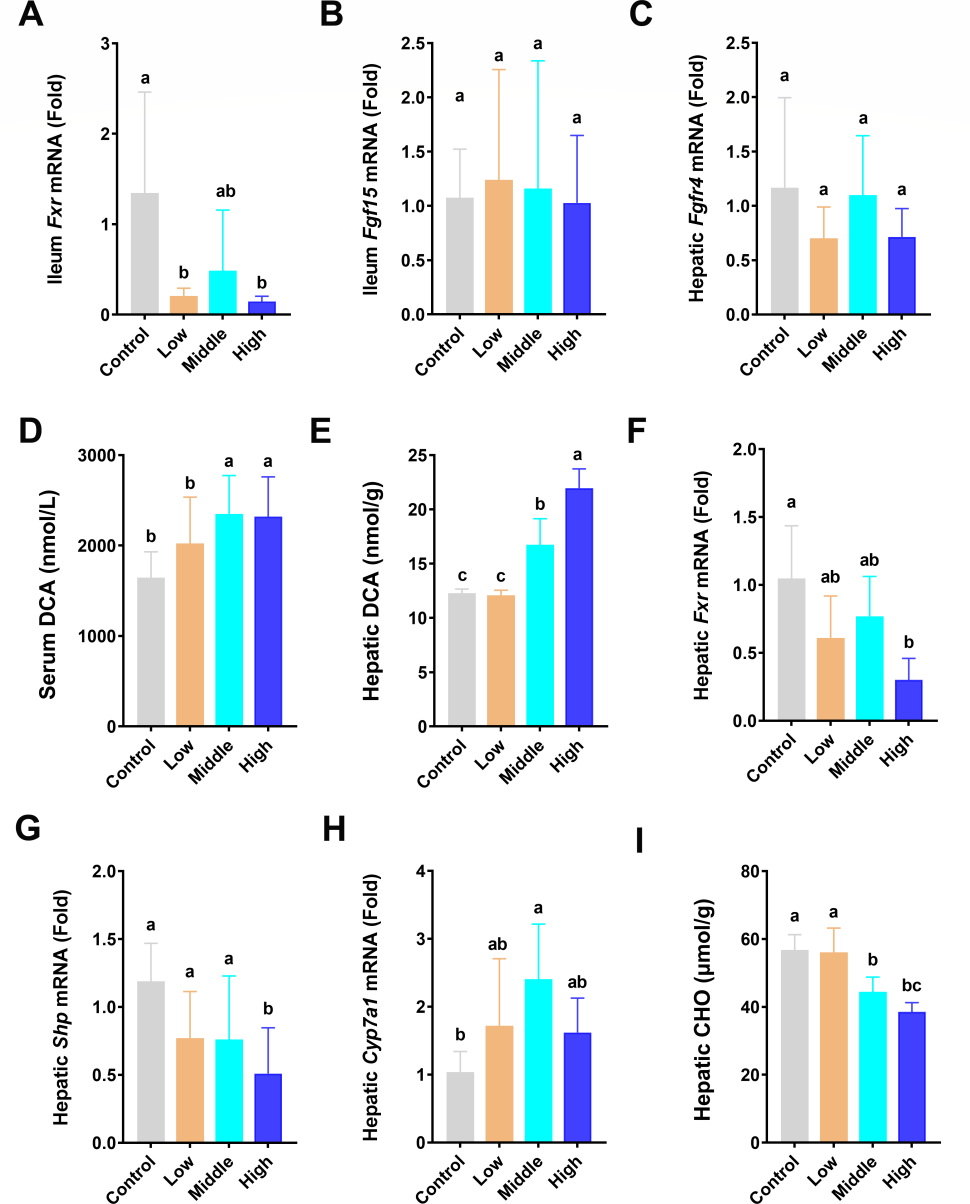
Disturbance of the *Fxr-Shp* signaling pathway by coumarin. (**A**) The mRNA level of *Fxr* in the ileum. (**B**) The mRNA level of *Fgf15* in the ileum. (**C**) The mRNA levels of *Fgfr4* in the liver. (**D**) The level of DCA in the serum. (**E**) The level of DCA in the liver. (**F–H**) The mRNA levels of *Fxr*, *Shp,* and *Cyp7a1* in the liver. (**I**) The level of CHO in the liver. Data are the means ± SD (*n* = 8) using one-way ANOVA. Different letters above the bars indicate significant differences between groups at the *P* < 0.05 level.

Compared with the control group, the *Fxr* in the ileum was markedly decreased in the low and high dose groups (Fig. 6A); however, the different doses of coumarin exhibited no significant change in the mRNA expression of the *Fgf15* in the ileum and *Fgfr4* in the liver (Fig. 6B and C). The serum DCA and hepatic DCA in the middle and high dose groups exhibited a notable elevation compared with the control and low dose groups (Fig. 6D and E). There was a significant decrease in the mRNA expression of hepatic *Fxr* and *Shp* in the high dose of coumarin (Fig. 6F and G). Hepatic *Cyp7a1* mRNA expression was remarkably increased in the middle dose group compared with the control group (Fig. 6H). The middle and high doses of coumarin significantly decreased the level of cholesterol (CHO) in the liver (Fig. 6I). Combining the results from Fig. 4F, the hepatic TBA in the middle and high dose group was markedly enhanced, suggesting that coumarin elevated hepatic TBA level by inhibiting the Fxr-Shp signaling pathway in the liver.

## DISCUSSION

*M. officinalis*, as a high-quality forage plant, is crucial for animal husbandry. However, *M. officinalis* contains coumarin, which has raised concerns about its safety. Although coumarin has some medicinal value, it can cause liver toxicity, which cannot be ignored. Studies have indicated that coumarin is metabolized in the liver primarily by the cytochrome P450 (CYP450) enzyme system (37). Metabolites of coumarin, particularly 3,4-epoxide intermediates, are associated with hepatotoxicity (7). Although the phenomenon of liver toxicity caused by coumarin has been observed, the specific mechanism still needs to be further studied. Here, this study investigated coumarin’s effects on intestinal permeability and gut bacterial composition, especially the abundance of *A. muciniphila*, LPS biosynthetic genes in the gut, hepatic oxidative stress response, and BAs metabolism. These results jointly showed that coumarin intake induced systemic inflammation and liver inflammation in mice.

The interaction between gut microbes and the host intestinal mucosal barrier is critical for the occurrence of host inflammation, as it is the first line of defense against harmful microbes and toxic substances (38). In this study, although α-diversity was not significantly altered, β-diversity analysis revealed a clear shift in microbial composition following coumarin treatment, indicating a disruption in microbial community structure. Among the differentially abundant taxa, *Coriobacteriaceae bacterium*, *Lachnospira pectinoschiza*, *A. muciniphila*, *Desulfovibrio brasiliensis*, and *Veillonella ratti* emerged as key responders to coumarin exposure (Fig. 2D). *Coriobacteriaceae bacterium* was identified as the top-contributing core microbe in the random forest model for predicting sample grouping (Fig. 2D), whose abundance was increased in the medium and high doses of coumarin. Previous studies have reported that *Coriobacteriaceae* may impair gut barrier integrity by downregulating molecules associated with tight junction function (39). *Desulfovibrio brasiliensis*, a sulfate-reducing bacterium, is known to produce hydrogen sulfide (H₂S), which may impair epithelial cell metabolism and disrupt barrier function (40). Our findings revealed that coumarin simultaneously expanded the populations of *Lachnospira pectinoschiza* and *Veillonella ratti*; however, the causal mechanisms linking these microbial shifts to barrier dysfunction remained to be elucidated. Among the significantly altered microbial taxa, we particularly focused on *A. muciniphila*, a well-recognized “next-generation probiotic” known for its critical role in regulating gut microbiota and intestinal barrier function (41). In microbiome studies, although genus *A. muciniphila* is relatively low in abundance in some samples, it has important biological functions and potential health effects, making it one of the focuses of research. Previous studies have demonstrated that *A. muciniphila* can enhance intestinal barrier integrity and increase the number of colonic goblet cells (42). Consistently, our results showed a significant positive correlation between the abundance of *A. muciniphila* and the number of colonic goblet cells (Fig. 2G). Coumarin markedly decreased the number of colonic goblet cells (Fig. 1B), suggesting the increased intestinal permeability (20, 21). Recent studies have highlighted its influence on barrier function through various mechanisms (43).

Tight junction proteins play a crucial role in maintaining the intestinal barrier function. *ZO-1* is the first tight junction protein to be identified, and its downregulation impairs the defense barrier function of intestinal mucosa (44). *Claudin 1* is a protein that regulates epithelial function, and its upregulation enhances the intestinal epithelial barrier (45). *Occludin* is also a widely used marker of intestinal barrier function, and the overexpression of *occludin* has been found to improve epithelial barrier function (46). The downregulation of these tight junction proteins leads to an increase in intestinal permeability (47), allowing the bacterial translocation and the passage of harmful substances across the intestinal barrier (48, 49). Notably, high doses of coumarin remarkably decreased the mRNA expression of *Occludin*, and the middle and high doses of coumarin significantly decreased the mRNA expression of *ZO-1* (Fig. 1D and E), indicating that coumarin can disrupt the intestinal barrier by downregulating the gene expression of key tight junction proteins, especially *ZO-1*. Immunofluorescence analysis further exhibited that the density of ZO-1 was significantly reduced in the middle and high dose groups (Fig. 1G). In addition, *Ptprh* is a key marker of intestinal permeability associated with the function of the intestinal barrier integrity (50). Our results exhibited that higher mRNA level of *Ptprh* was observed in middle and high doses of coumarin (Fig. 2H). Overall, our findings indicated that coumarin treatment disrupted intestinal permeability.

LPS is a part of the outer membrane of gram-negative bacteria, which can activate a variety of cells related to inflammation, such as monocytes and macrophages, causing systemic inflammation (51). LPS has been reported to pass from the gastrointestinal tract into the bloodstream, triggering or enhancing a variety of diseases, including systemic inflammation (52). The upregulation of serum LPS level is directly caused by intestinal barrier dysfunction (53). In our study, LPS biosynthesis genes in the gut microbiota were significantly increased after treatment with the middle and high doses of coumarin, resulting in the markedly elevated serum LPS level (Fig. 3A and B). The significant increase in serum IL-6 and IL-1β (Fig. 3C and D) further supported that coumarin can cause systemic inflammation by elevating the pro-inflammatory cytokines, which can play a central part in the initiation and amplification of the inflammatory response (54). The latest DSS-induced colonic barrier disruption was observed in mice, with serum LPS increasing by 3–4 times, accompanied by upregulation of IL-6 and IL-1β (55), which was similar to the results of this study.

Systemic inflammation frequently coexists with hepatic inflammation. We found that the middle and high doses of coumarin caused the infiltration of hepatic inflammatory cells following systemic inflammation (Fig. 4E). Liver inflammation can cause an increase in oxidative stress response. In our study, middle and high doses of coumarin significantly increased hepatic SOD and T-AOC activities (Fig. 4C and D). SOD is an important antioxidant enzyme that can protect the body from oxidative stress damage by eliminating harmful free radicals (56). T-AOC reflects the overall antioxidant capacity of the liver (57). The increase in SOD indicates an increase in the production of reactive oxygen species (ROS) in the liver, and the accumulation of ROS can attack cell membranes, proteins, etc., leading to hepatocyte damage and aggravation of the inflammatory response (58). The increase of T-AOC reflects the body’s resistance to oxidative stress in the inflammatory state, but this resistance may not be sufficient to completely eliminate the oxidative damage (59). Instead, it may promote the release of inflammatory factors, thus aggravating the hepatic inflammation.

BAs, as important substances secreted by the liver, play a crucial role in inflammation (60). In this study, coumarin altered the composition of the BA pool; the liver TBA in the middle and high dose groups was remarkably enhanced (Fig. 4F), and the serum TBA in the high dose group was markedly elevated (Fig. 4G). The BAs in the serum and liver of the control group revealed that the conjugated BAs (TCA, T-β-MCA, and T-α-MCA) were the most prevalent in the liver BA pool (Fig. 5A), which was in line with the typical BA composition in mice (61). The marked elevation of the conjugated BAs (DCA and CA) in the middle and high doses of coumarin (Fig. 5C) indicated the alteration of BA composition and BA metabolism in the liver. In the serum, the significant reduction of conjugated BAs (TCDCA) in the high dose group and the significant increase of unconjugated BAs (DCA and CA) in the middle and high dose groups (Fig. 5D) suggested that coumarin may disrupt the serum BA balance and BA signaling. Previous studies have shown that highly hydrophobic BAs, such as DCA and CA, possess strong cytotoxicity, and DCA has been found to trigger multiple inflammatory factors *in vitro* (62). Another study reported that CA exacerbated hepatic inflammation by increasing mRNA transcripts of IL-1β and IL-6 and accelerating fibrosis development in DKO mice compared with baseline (63). These studies further support the significant role of highly hydrophobic fatty acids (DCA and CA) in the inflammatory mechanism of coumarin-treated mice.

FXR is a nuclear receptor activated by BAs in enterohepatic circulation (64). FXR plays a key role in BA metabolism. In the liver, BAs-activated *Fxr* can induce the expression of *Shp*, which can combine with hepatic receptor homology 1, thereby inhibiting *Cyp7a1* gene expression in the liver (65). *Fxr* activated by BAs in the ileum induces the expression of *Fgf15*, which enters the liver via portal vein blood and combines with FGFR4, triggering the JNK1/2 and ERK1/2 signaling cascade and further inhibiting *Cyp7a1* expression (66). Normally, the activation of *Fxr* in the liver suppresses the expression of the *Cyp7a1* gene, thereby controlling the rate of BAs synthesis (67). Activation of *Fxr* can reduce the synthesis of BAs by upregulating the expression of *Shp*, thus reducing the toxicity of BAs (68). When the *Fxr-Shp* signaling pathway is inhibited, the synthesis of BAs may increase, leading to the accumulation of BAs in the liver. In our study, the decreased expressions of *Fxr* and *Shp* in the liver may lead to a loss of this inhibitory control, elevating the expression of *Cyp7a1* and resulting in the increase of TBA. There is a lot of evidence that the elevated TBA levels are a marker of liver disease, including liver inflammation (69). In cholestatic liver disease, the elevated levels of BAs lead to liver cell damage and an inflammatory response (33). By inhibiting the *Fxr-Shp* pathway, LPS can enhance BA metabolism and increase liver toxicity (70), which is similar to the results of our study. *Fxr* has been identified as a key target for the treatment of liver injury, cholestasis, and chronic inflammatory diseases (71). *Fxr* can strongly induce the expression level of *Shp*, and *Shp* can reduce liver inflammation (72). In addition, knockout of *Shp*-related genes can lead to increased liver inflammation and necrosis (73). Therefore, the *Fxr-Shp*-signaling pathway is critical for BA regulation and the occurrence of liver inflammation. This study further supported the finding that coumarin induced liver inflammation by regulating the *Fxr-Shp* signaling pathway, especially in the high dose group. This study provides comprehensive insights into the multifaceted effects of coumarin on intestinal permeability, gut microbiota, bile acid metabolism, and hepatic inflammation. Future research should focus on long-term exposure conditions and potential therapeutic strategies targeting bile acid signaling to mitigate the adverse effects of coumarin. Moreover, the research is conducted in mice, and further studies are needed to confirm whether similar effects occur in ruminants.

### Conclusion

**Fig 7:**
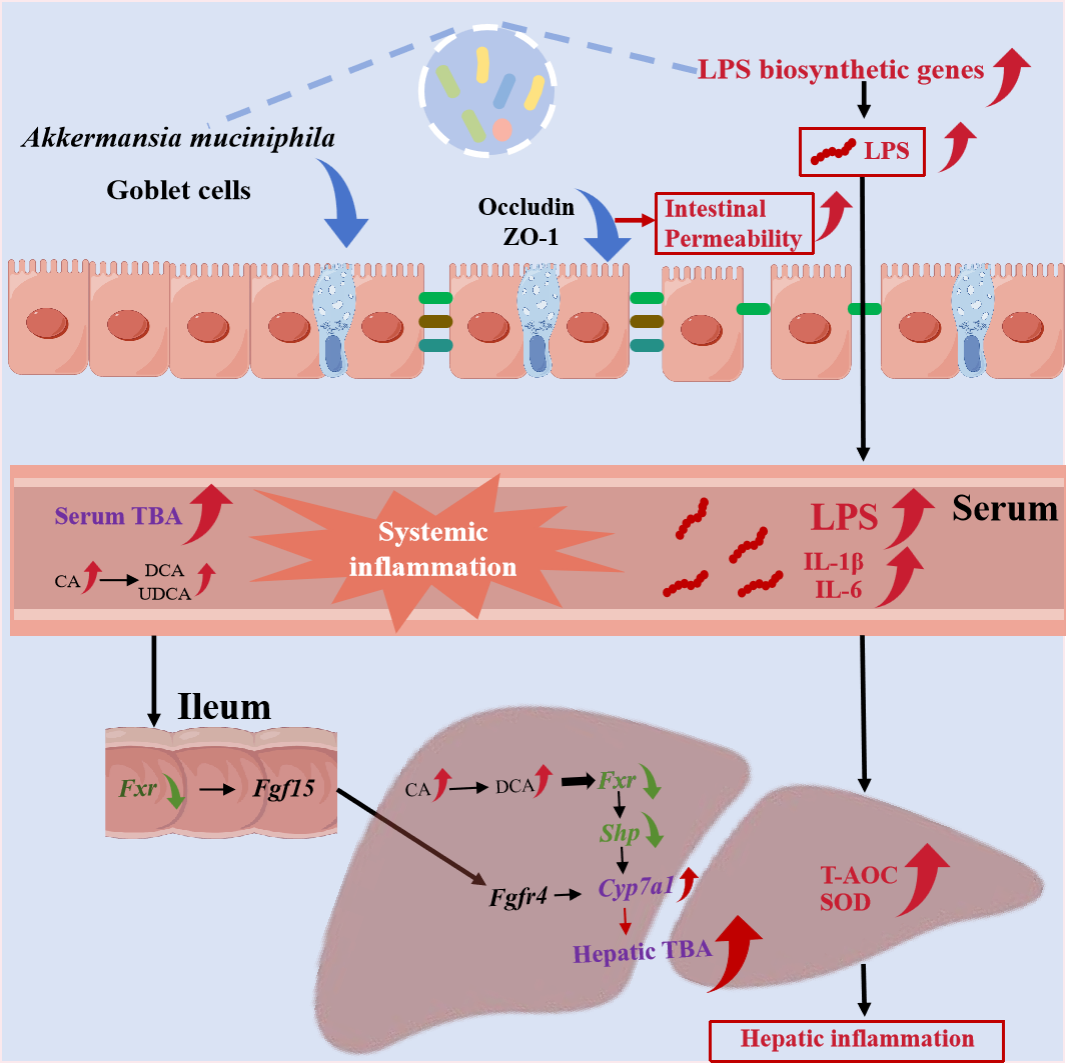
The mechanism linking the intestinal permeability and bile acid accumulation via inhibition of the FXR-SHP pathway to coumarin-induced systemic inflammation.

In conclusion, coumarin significantly increased intestinal permeability by reducing beneficial intestinal bacteria such as *Akkermansia muciniphila* and disturbing intestinal barrier function, as evidenced by the reduced tight junction protein (ZO-1) and fewer colonic goblet cells. It is notable that the elevated LPS biosynthesis in gut microbiota caused higher serum LPS level and systemic inflammation, exacerbating oxidative stress and elevating TBA level in the liver. Additionally, coumarin inhibited *Fxr* and *Shp* expressions in the liver, thereby promoting *Cyp7a1* transcription and upregulating TBA level in the serum and liver. Overall, these findings highlight the critical role of gut-liver axis disruption in coumarin-induced inflammation and provide valuable insights into its hepatotoxic mechanisms (Fig. 7).

## Data Availability

The metagenomic sequencing data of all samples were deposited into NCBI Sequence Read Archive (SRA) database under the accession number PRJNA1074524.

## SUPPLEMENTAL MATERIAL

Supplemental material spectrum.01415-25-s0001.docx Fig. S1; Tables S1 and S2.

ASM does not own the copyrights to Supplemental Material that may be linked to, or accessed through, an article. The authors have granted ASM a non-exclusive, world-wide license to publish the Supplemental Material files. Please contact the corresponding author directly for reuse.
