# Erratum for Shou et al., “Increased intestinal permeability and bile acid accumulation via inhibition of the FXR-SHP pathway contribute to coumarin-induced systemic inflammation”

**DOI:** 10.1128/spectrum.01054-26

**Published:** 2026-07-10

**Authors:** Na Shou, Dandan Wu, Qi Wang, Ping Huang, Qiwen Lin, Senao Hou, Keyi Fu, Wenqian Xu, Jiyu Zhang, Zunji Shi

## ERRATUM

Volume 13, no. 11, e01415-25, 2025, https://doi.org/10.1128/spectrum.01415-25. In the Acknowledgments and Funding sections, the grant number from the National Natural Science Foundation of China should read “U23A20216” instead of “U23A200206.”

