# Supplementary material for: Increased intestinal permeability and bile acid accumulation via inhibition of the FXR-SHP pathway contribute to coumarin-induced systemic inflammation
Source: Microbiol Spectr. 2025 Sep 19;13(11):e01415-25. doi: 10.1128/spectrum.01415-25 (PMC12584623; doi:10.1128/spectrum.01415-25)
Supplement: Supplemental material — Fig. S1; Tables S1 and S2. [file spectrum.01415-25-s0001.docx]

**Increased intestinal permeability and bile acid accumulation via inhibition of FXR-SHP pathway contribute to coumarin-induced systemic inflammation**

Na Shou^1^, Dandan Wu^1^, Qi Wang^1^, Ping Huang^1^, Qiwen Lin^1^, Senao Hou^1^, Keyi Fu^1^, Wenqian Xu^1^, Jiyu Zhang^*,1^ and Zunji Shi^*,1^

^1^ State Key Laboratory of Herbage Improvement and Grassland Agro-ecosystems, Center for Grassland Microbiome, College of Pastoral Agriculture Science and Technology, Lanzhou University, Lanzhou, 730000, China

* Corresponding author: Dr. Zunji Shi, Tel: 86-0931-8913074; Fax: +86-0931-8913074; Email: [shizj@lzu.edu.cn](mailto:shizj@lzu.edu.cn); Dr. Jiyu Zhang, Tel: 86-0931-8913074, Fax: +86-0931-8913074, Email: zhangjy@lzu.edu.cn

Table S1. Primers used for quantitative real-time PCR.

|  | Forward primer (5’ -3’) | Reverse primer (5’-3’) |
| --- | --- | --- |
| *Fxr* | TCCAGGGTTTCAGACACTGG | GCCGAACGAAGAAACATGG |
| *Shp* | CGATCCTCTTCAACCCAGATG | AGGGCTCCAAGACTTCACACA |
| *Cyp7a1* | AGCAACTAAACAACCTGCCAGT | ACTAGTCCGGATATTCAAGGATGCA |
| *Fgf15* | ACGTCCTTGATGGCAATCG | GAGGACCAAAACGAACGAAATT |
| *Fgfr4* | CTGTATGGGCTAATGAGGGAGTG | TCAGGCGGAGGTCAAGGTAC |
| *β-actin* | GTACCACCATGTACCCAGGC | AACGCAGCTCAGTAACAGTCC |
| *Ptprh* | *GGTAAAAGTGGGTAGGAAATGGC* | *GTGGCTGTGTAGGACTGAGC* |
| *A. muciniphila* | TTGAAGAAGAGCCCATCCTC | CAGCTCATATGGGTCCGAC |
| *Claudin 1* | CAGATATGAATTTGGTCAGGCTC | CACTGGAAGGCGAAGGTTT |
| *Occludin* | TCTCAGCCAGCGTATTCTTTC | GCACATCACGATAACGAGCAT |
| *ZO-1* | GGGGCAATCTCAACTCCTGT | GGTTGTCCAACTTGGGCAT |

*Fxr*: farnesol X receptor; *Shp*: small heterodimer partner; *Cyp7a1*: cholesterol 7-alpha hydroxylase; *Fgf15*: fibroblast growth factor; *Fgfr4*: fibroblast growth factor receptor. *Ptprh*: protein tyrosine phosphatase receptor type H; *A. muciniphila*: *Akkermansia muciniphila*.

Table S2. The full names and classifications for 28 bile acid standard compounds.

| Abbreviation | Full Name | Groups |
| --- | --- | --- |
| T-α-MCA | Tauro-alpha-Muricholic acid | Conjugated |
| T-β-MCA | Tauro-beta-Muricholic acid | Conjugated |
| TCA | Taurocholic acid | Conjugated |
| THCA | Taurohyocholic acid | Conjugated |
| TDCA | Taurodeoxycholic acid | Conjugated |
| TLCA | Taurolithocholic acid | Conjugated |
| TUDCA | Tauroursodeoxycholic acid | Conjugated |
| TCDCA | Taurochenodeoxycholic acid | Conjugated |
| GLCA | Glycolithocholic acid | Conjugated |
| GDCA | Glycodeoxycholic acid | Conjugated |
| GCDCA | Glycochenodeoxycholic acid | Conjugated |
| GHDCA | Glycohyodeoxycholic acid | Conjugated |
| CA | Cholic acid | Unconjugated |
| DCA | Deoxycholic acid | Unconjugated |
| LCA | Lithocholic acid | Unconjugated |
| HCA | Hyocholic acid | Unconjugated |
| ACA | Allocholic acid | Unconjugated |
| GCA | Glycocholic acid | Unconjugated |
| CDCA | Chenodeoxycholic acid | Unconjugated |
| HDCA | Hyodeoxycholic acid | Unconjugated |
| UDCA | Ursodeoxycholic acid | Unconjugated |
| nutriCA | Nutri cholic acid | Unconjugated |
| iso-DCA | Iso-Deoxycholic acid | Unconjugated |
| 12-ketoDCA | 12-keto deoxycholic acid | Unconjugated |
| iso-LCA | Iso-lithocholic acid | Unconjugated |
| α-MCA | Alpha-Muricholic acid | Unconjugated |
| β-MCA | Beta-Muricholic acid | Unconjugated |
| ω-MCA | Omega-Muricholic acid | Unconjugated |


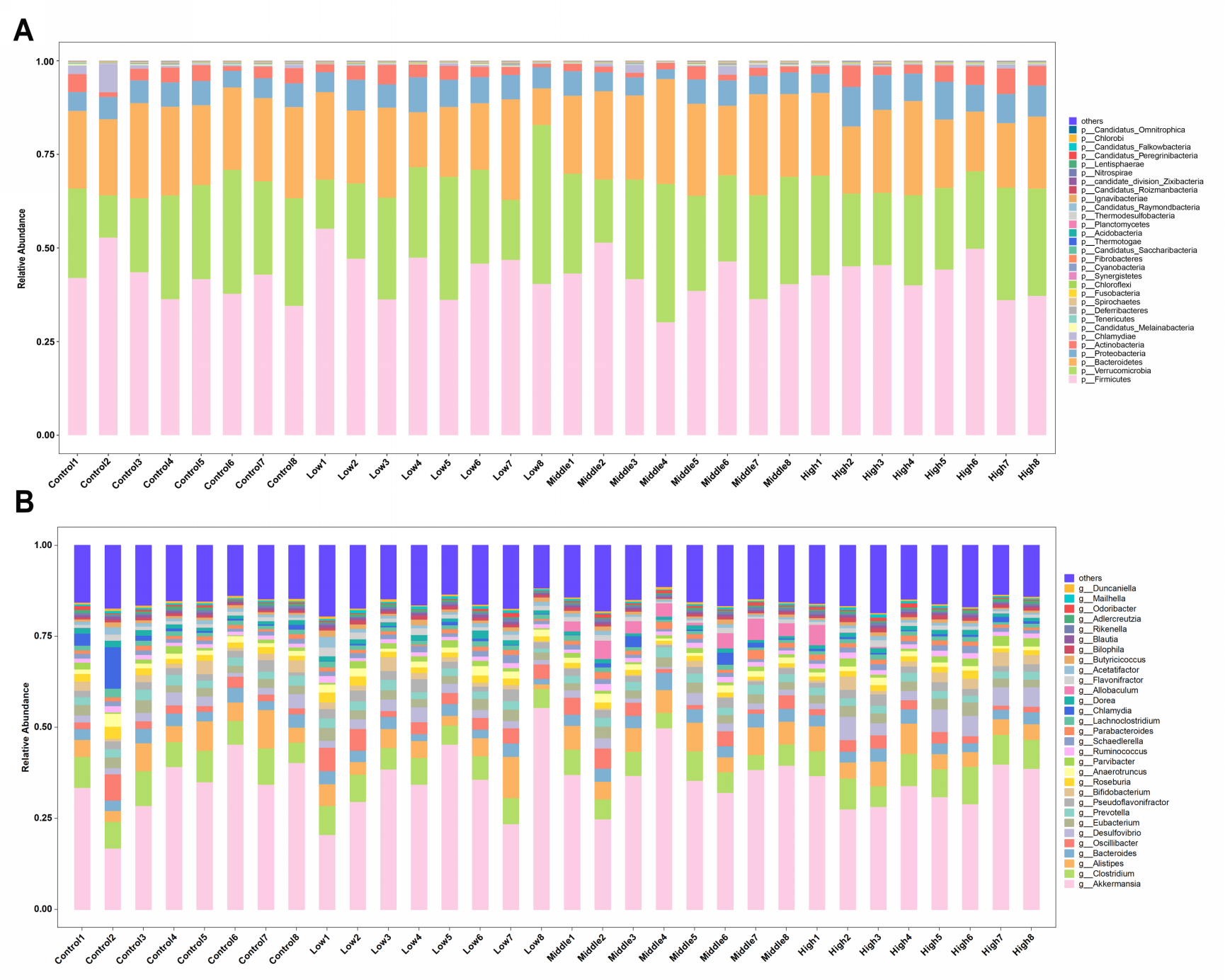


**Fig.S1** The abundance of bacterial by metagenomic analysis. (A) The abundance of bacteria at phylum level. (B) The abundance of bacteria at genus level.
